# Policies and Challenges on the Distribution of Specialists and Subspecialists in Rural Areas of Iran

**DOI:** 10.3390/medicina55120783

**Published:** 2019-12-13

**Authors:** Seyed Masoud Mirmoeini, Seyed Sina Marashi Shooshtari, Gopi Battineni, Francesco Amenta, Seyed Khosrow Tayebati

**Affiliations:** 1 Virtual School, Tehran University of Medical Sciences, Tehran 1417466191, Iran; dr.mirmoeini@yahoo.com (S.M.M.); sina.marashi@gmail.com (S.S.M.S.); 2 School of Medicinal Sciences and Health Products, University of Camerino, 62032 Camerino, Italy; gopi.battineni@unicam.it (G.B.); francesco.amenta@unicam.it (F.A.)

**Keywords:** telehealth, far-away areas, physician distribution, health policies, specialist and subspecialist

## Abstract

*Background and objectives:* Having fair access to medical services may probably be a standard feature and indisputable right of all health policies. The health policy of Iran enunciates this right. Unfortunately, as may happen in many countries, the execution of this policy depends on different factors. Among these parameters, the suitable distribution of professionals, hospitals, and medical facilities should be quoted. On the other hand, in Iran, there are many other problems linked to accessing areas with natural hindrances. *Materials and methods:* A literature search was conducted in PubMed and CINAHL libraries, specifically studies from 2010 to 2019. A Boolean operated medical subject headings (MeSH) term was used for the search. Newcastle–Ottawa Scale (NOS) scoring was adopted to assess the quality of each study. *Results:* A total of 118 studies were displayed, and among them, 102 were excluded due to duplication and study relevance. Study selection was made based on content classified into two groups: (1) shortage and unsuitable distribution of specialist and subspecialist physicians in Iran and (2) studies that explained the status of degradation in different areas of Iran. Outcomes demonstrated that Iran is generally suffering a shortage and unsuitable distribution of specialists and subspecialists. This lack is particularly crucial in deprived and areas far away from the cities. *Conclusions:* The present study analyzed in detail research studies regarding policies and challenges that reflect on the provision of specialists and subspecialists in Iranian rural areas.

## 1. Introduction

The issue of justice in the distribution of the specialist medical workforce is at the center of attention in most countries. It is necessary to prepare the basics for having justice in the human resources distribution [1]. The number of workers in the medical system of different countries on the one hand, and fair distribution of medical services in various areas of a country in compliance with its demographic, epidemiologic, and etiologic properties on the other, are aspects that should be considered [2]. 

In a study that was performed in order to describe a method of supplying and distributing active clinical physicians in the USA in 2005 by focusing on rural areas, an unbalanced distribution of physicians between cities and villages and a diverse distribution of physicians in rural areas were observed, and the total ratio of specialist/population in rural areas was small [3]. Moreover, native and non-native workforces, at equal rates, asked to be transferred from disadvantaged areas. This trend has demonstrated that the policies of educating indigenous specialist forces has not lead to maintaining specialist physicians in deprived areas [3,4]. Only 9% of physicians work in nonurban areas, where 20% of the USA population lives [5]. Likewise, persons residing in rural areas have equal rights to benefit from high-quality medical services as persons living in cities. 

The World Health Organization (WHO) believes that public access to motivated, active, and skillful medical workforces, especially in rural areas, is the necessary condition to better understand medicine, human rights, and social justice [6]. In 2010, support for patients and cost-effective medical care were implemented for all US citizens. However, it is not possible to reach the goals of this law until there is a balance between offering and using global medical services [6,7]. Traveling to a specialty center is problematic for families since it disturbs their regular daily schedules, education, and leads to absence from the workplace for adults [8]. The impediments created for rural societies have led to different medical care for children. These imbalances are caused by the low access of newborns and children to health services. According to the census in 2010, nearly 20% of the USA population resides in rural areas; thus, many children have to face shortages for obtaining important health and medical services [9]. These hindrances include geography, relative scarcity and unsuitable distribution of general and subspecialty pediatricians, and socioeconomic impediments for traveling. These obstacles may endanger children’s health. General practitioners that work in villages often have barriers to accessing the subspecialty of pediatrics.

In the Iranian health system, there is a strong focus on improving the quality of health services and access to them, in particular for deprived social classes [10]. Physicians are unsuitably distributed in different cities, and there are a limited number of physicians in rural areas. The absence of enough physicians in rural areas represents a severe problem for the Iranian Health Ministry. In general, the health of rural people benefits from the development strategy below [11]. Access to medical services depends on many factors; nevertheless, the most fundamental one is the presence of operators offering medical support. Overall, the main problem in rural areas of Iran is an unsuitable distribution of physicians. As of this, the present work analyzes primary studies that report specialty and subspecialty challenges facing Iran in the distribution of medical services. The advantages of telehealth and the uncertain limitations of telehealth practice are also critically discussed. 

## 2. Methods

### 2.1. Data Sources and Study Selection

**Figure 1:**
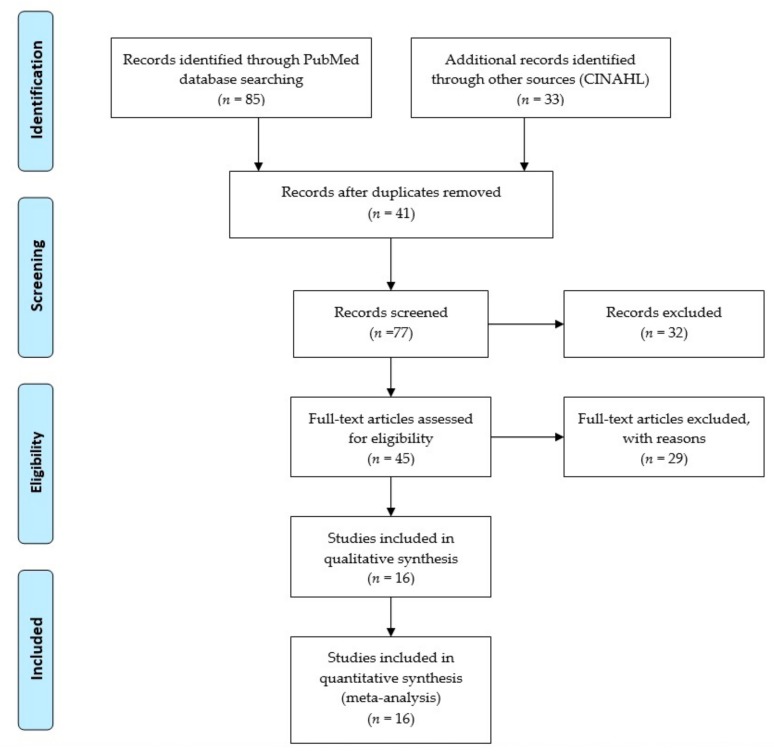
PRISMA—transparent reporting of systematic reviews and meta-analyses [13].

We considered studies related to policies and challenges in the distribution of medical experts, mainly in the rural areas of Iran. A literature search was conducted in the two libraries of PubMed (Medline), Cumulative Index of Nursing and Allied Health Literature (CINAHL) with medical subject headings (MeSH). The search terms included “polices and challenges health provision of rural areas” OR “challenges in distribution of specialist and subspecialist services” OR “studies related status of degradation in Iran rural areas”. The search process is illustrated in Figure 1. Search outcomes produced 118 studies.

### 2.2. Quality Assessment 

The quality check of each study was done by the Newcastle–Ottawa Scale (NOS) [12]. Each study quality assessment was outlined as weak (0–4), moderate (5–6), or strong (7–9). The remaining 16 studies in line with study objectives were further analyzed in detail. 

### 2.3. Exclusion Criteria

Studies or other material sources that were published before 2010 were excluded from our study because our study aimed to cover the latest trends in policies and challenges associated with the medical practitioner distribution of Iran. Review articles and studies that did not address the issues of specialist and subspecialist care in rural areas of Iran were excluded from the study. A total of 102 studies were excluded after considering the availability of full text, duplications, topic relevance, language concerns, and NOS satisfaction rates.

## 3. Results

The selected studies based on content were classified into two groups: (1) policies and unsuitable distributions of specialist and subspecialist physicians in Iran and (2) studies that explained the status of degradation in different areas of Iran. The key findings of different studies are presented below. The main characteristics of each study (first author, year, study type, objective, policies, and challenges) are summarized in tabular format (Table 1 and Table 2) per each group of analysis. 

### 3.1. Policies and Specialist Distribution for Pediatric Assistance 

#### 3.1.1. Problems in Specialist Consultation 

Equal distribution of medical resources and its influence on the quality and quantity of offered services has challenged medical policymakers. On the other hand, in the Iranian health system, the presence of specialists in different fields, including internal medicine, pediatrics, gynecology, and obstetrics, is mandatory. The health system suffers an acute shortage of specialists in orthopedics, anesthesia, neurosurgery, ophthalmology, radiology, gynecology, otolaryngology, and general surgery, and training staff in these specialties is not feasible in the short run. Actually, in Iran, this gap has mainly depended on the presence of 40% of medical graduates in Tehran, which has led to a shortage of physicians in other areas [20]. Unfortunately, in Iran, there has been no study performed in the field on the distribution of the pediatrics subspecialty.

Another limitation for medical practitioners in Iran is that most of the physicians do not have an idea of primary immunodeficiency diseases (PIDs). In the study of [19], there is an extended absence of awareness of PIDs among doctors. This might be one of the significant reasons for late consultations and the postponement of satisfactory treatment leading to patient morbidity and mortality. Retraining classes and re-examining teaching timetables are required as a practical methodology to improve doctor awareness of PIDs [19]. Nevertheless, according to a report published in 2016, only 25 pediatric rheumatologists are active in Iran, especially in Tehran, Alborz, and Khorasan Razavi regions [22]. Therefore, other patients do not have the opportunity to visit a pediatric rheumatologist, and they have to be referred to an adult rheumatologist and even face wrong diagnoses. 

In the study of [21], despite the acceptable growth of different medical groups in the last two decades, an unbalanced physician placement has prevented enjoying this development and promotion. Overall, in Iran, the distribution of general practitioners, specialists, and subspecialists is unstable and may lead to negligence in needy areas. To overcome these issues, health technology assessments (HTAs) have been built up in the human services arrangement of Iran. However, constant political will is required to push forward the targets of HTA in Iran. Like in other countries, this will advance the guidelines on the selection of new medical services to improve specialization and productively distribute wellbeing [14]. 

#### 3.1.2. Expenditure and Medical Policies to Supply Medication 

Despite this, a study [15] on the expenditure of drug supply to Iranian urban households mentioned that medications are nonflexible and fundamental for families. Health policymakers in Iran should develop proper planning to guarantee both the physical and money-related openness to drugs by urban families. The advancement of fundamental and valuable medical coverage, particularly for poor populations and urban territories where there are patients with ceaseless illnesses, can be a suitable solution for reducing barriers to securing necessary medications [15]. At the same time, providing proper statistical methods and models to break down information is undoubtedly essential to work around issues and acquire valid model parameters to produce an accurate assessment of medical costs [18]. 

#### 3.1.3. Factors that Influence the Geographic Distribution of Iran Physicians 

It appears that the local distribution and supply of doctors has been improved in the recent policy implementation [17]. Different factors influence doctor decisions to remain and practice in private and rural areas [23]. In a study on health disparities of Iranian woman [16], determinants like medical behavior, medical knowledge, and lifestyle availability were adopted and showed the imbalance in women’s health at a provincial level. Therefore, health policymakers to provide better medical services should address these disparities in determinants. 

### 3.2. Health Status Degradation in Different Areas of Iran 

Regardless of human wealth and personal assets, Iran still experiences elevated levels of disparity and neediness. It is one of the progressing nations where there are still huge contrasts between areas. A study performed in 2017 classified Iran’s provinces based on their development index. Eleven regions (43.85%) were among the disadvantaged, eight areas (33.3%) were considered developed, and Tehran, Isfahan, and Fars were highly developed provinces [24]. In 2013, an evaluation of the development level in different Iranian big cities (216) demonstrated that the level for most of them (64%) was lower than the medium level. Among these 216 cities, 95 were classified as relatively disadvantaged, 85 as underprivileged, and 36 cities were categorized as very disadvantaged. Study findings suggested that poor implementation policies were affecting the urban planning systems of Iran [25].

In further research, 30 Iranian provinces were examined from the viewpoint of their access to medical care services. The results of this research evidenced that among these 24 provinces, there were ten developed, seven semi developed, and seven underdeveloped [26]. Moreover, another study showed that 64.2% of Iranian cities were among underdeveloped areas [30].

According to the studies performed in the Markazi province in Iran, there are high imbalances in the dispersion of medical service facilities in the Markazi area. Researchers suggest that policymakers decide resource allocation needs as indicated by the level of improvement for appropriate and equivalent dissemination of medicinal service facilities [27]. Study outcomes of [28] mentioned that substantial financial imbalances were seen in various wellbeing areas for gatherings of better financial status. Because of these outcomes, policymaking planned for handling disparities should focus on various wellbeing spaces just as on overall health. 

Another retrospective study [29] on the resource distribution between Iran provinces in 2014 mentioned that the dissemination of health policy assets between Iran areas was equivalent. In this manner, health policymakers need to center on different parts of availability, for example, circulation of assets as indicated by health needs, distribution of resources within the provinces, and quality of service provisions. 

## 4. Discussion

We aimed to review studies that reflected the policies and challenges on the distribution of medical practitioners in rural areas of Iran. The distribution of medical experts represents a big challenge for many countries. This problem was more evident in countries with large areas covered with natural hindrances, like mountains and deserts or islands [31]. In these countries, governments are significantly challenged to resolve these problems. It is clear that the resolution of these problems is hard for anyone, but it is probably more difficult for the nations that are poor. Luckily, Iran is not among these countries, and every year several new doctors start their activities; however, it looks like this is not enough. Statistical data has demonstrated that more than 40% of doctors reside in Tehran and desire to work there. This kind of choice unavoidably leads to an essential discrepancy between cities and villages. Therefore, for governments to fill this gap, they will need to implement some winning strategies to make attractive those places that are not. 

Apart from some particular strategies to improve the attraction to serve in disadvantaged areas, a technologically advanced service may be implemented. In particular, different kinds of medical assistance can be supplied for children by telehealth or telemedicine [32]. The pediatric population needs many types of health services. They should be periodically checked for their growing process, for correct dental implantation, for different disorders related to their eyes and so on [32,33]. Furthermore, it is also possible to add to this basic help to other medical problems (e.g., cardiac malformations, respiratory allergy, etc.). Therefore, the presence of a specialist, at least in a virtual modality, may provide crucial aid to this population. Telehealth really can support them using technological health devices. These could collect crucial medical information and transmit it to the specialists that can supply a first or second opinion [34]. Implementation of these facilities needs suitable infrastructures even if some services may be provided using videoconferencing or emails. 

Telehealth is considered an electronic communication technology for offering health and medical services to people who live in internal areas. It is very important because of the long distance between the physician and patient [35]. However, the most “natural” application of telemedicine would be the ability to use teleconferencing for communicating with patients and health specialists. Telemedicine is viewed as a potential solution for coping with the shortage of specialists in India [36]. Different reports have confirmed the satisfaction of this technology for both patients and physicians [37]. 

In general, people residing in rural areas that require specialized medical care face challenges in receiving specialty and subspecialty medical services because they need repeated medical evaluations of their health [38]. Results of studies show it is possible to offer specialty and subspecialty telemedicine counseling for these people requiring specific medical care who live in rural and deprived areas [38,39]. This strategy leads to a high level of satisfaction among patients, supervisors, and persons offering medial services. Ninety-eight percent of patients or supervisors described that receiving telemedicine counseling services may be continued instead of traveling to several specialty centers to have face-to-face meetings [40]. Therefore, the use of telemedicine is an attractive recommendation for persons/centers offering subspecialty services of medical care [40,41]. It should be noted that many of these services are offered through video conferencing (synchronous), and the patient communicates with the person offering service. 

## 5. Conclusions

All studies mentioned above suggested that a tele medical strategy, using medical informatics and other advanced systems, may offer many advantages for people that live in underprivileged areas where the population tends to more vulnerable. On the other hand, the formation and distribution of medical experts (physicians, nurses, etc.) represent an essential challenge for all countries. This problem has significantly hit Iran. Many resources have been used to increase the number of health professionals, but most prefer to stay in big cities and are sparse in remote areas. However, this shortage of specialist and subspecialist services should be covered, and in particular, to private area patients, because there is an excellent pediatric population that needs both care and prevention services. Governments and health policymakers also need to take initiatives on the distribution of sources and allocation of funds in health care development especially for people living underprivileged areas. Nevertheless, the development and use of a telehealth system has become crucial to help these populations.

## Figures and Tables

**Table 1:** Study details associated with the polices and distribution of pediatric specialist assistance in Iran.

First Author	Year	Study Type	Objectives	Policies	Challenges
Doaee, S.H. [14]	2013	Policy study	Health technology assessments (HTAs)	Generating localization for fundamental HTAs in the Iranian health system. In medical universities, for M.sc, Ph.D. degrees, design policy in the HTA subject	There is no clear government policy to move forward with HTA objectives in Iran
Karami Matin [15]	2015	Analytical descriptive study	Finding the key factors that affect the expenditures of pharmaceutical products by municipal houses	For poor people, designing and developing primary and supplemental insurance coverage can be a suitable solution to avoid barriers of drug availability for patients with chronic diseases	Health policymakers in Iran are not ready to take steps to ensure physical and financial access to drugs
Bayati, M. [16]	2017	Original study	Framework for women’s health determinants	At a provincial level, determinants like health behavior, health knowledge, and lifestyle availability were imbalanced in women’s health at a provincial level	Too many provincial disparities of Iranian women were found. Health policymakers should address these disparities in determinants
Haghdoost, A. [17]	2010	Survey	Geographical distribution of specialists and medical experts and the inequalities in Iran provinces	The country is more specialized in different medical subgroups, and other provinces still require specialist medical personnel	Specialist group geographical distribution is unbalanced across the country, and there seems to be a significant shortage in deprived areas.
Parsaeian, M. [18]	2014	Original study	Provide remedies to overcome the burden of disease and geographical inequalities of Iran	Relating various available data sources and generating reliable and precise evidence for Iranian burden of diseases and its risk factors	Not identified
Nourijelyani, K. [19]	2012	Experimental	Assessment of the Iranian general practitioners (GPs) knowledge and pediatricians about primary immunodeficiency diseases (PIDs)	Reconsidering educating schedules and training classes is necessary to improve physician’s knowledge about PIDs.	There is an impressive absence of awareness of PIDs among doctors
Aeenparast, A. [20]	2012	Cross-sectional	Determine the waiting time in doctor workplaces in Tehran, Iran	The normal waiting time in this research was less than seven days for specialists and just about seven days for subspecialists	The Iranian health system has not set up a complete referral framework, and with this circumstance, waiting times may strongly affect patient health
Rashidbeygi, M. [21]	2013	Experimental	Finding the knowledge of physicians towards evidence-based medicine (EBM)	Information and frame of mind of young doctors were progressively founded on EBM and contrasted with old doctors	A huge contrast in the information mean score of doctors demonstrates that EBM is still new in Iran
Keley, E. [22]	2016	Cross-sectional	The connection between doctors’ attributes and their desire to practice in rural areas was analyzed	It appears that expanding the enrollment of specialists from a rural background in residency projects may overcome the issue of uneven dissemination of specialist physicians in Iran	Not identified
Ravaghi, H. [23]	2015	Qualitative	Investigate the factors affecting the distribution of expert doctors in Iran	The territorial distribution and supply of specialists of Iran have been improved in light of the executed policies in recent years	Students with a rural background and steady measures for doctors working in deprived regions were suggested

**Table 2:** Studies that explain the status of degradation in different areas of Iran.

First Author	Year	Study Type	Objectives	Policies	Challenges
Ghaderi, M. [24]	2017	Survey	Impact factors on the management of regional development in Iran	Produce solutions to reduce regional imbalances	Policies proposed in this study increase imbalances and threaten disorder for all of Iran
Rasoolimanesh, S. [25]	2013	Qualitative	Assessment of the existing urban planning system of Iran	Findings cause poor urban plan management	Poor implementation
S. Emamgolipor Sefiddashti [26]	2017	Cross-sectional	Ranking the countries based on access to health sector indicators	Produce a report on the need for specialists in Iranian provinces	Healthcare expenditure not yet successful in Iran
Abolhallaje, M. [27]	2014	Cross-sectional	In terms of access to health care, ranking the towns of the provinces was done	Policymakers should come forward to bridge the gap between the distribution of health services in Markazi	The large gap for healthcare provision in towns of Markazi province, Iran
Baigi, V. [28]	2018	Survey	Assessment of socioeconomic inequality in various health domains and self-rated health (SRH)	Socioeconomic inequalities were found in various health domains in favor of improved socioeconomic status groups	Not identified
Lotfi, F. [29]	2018	Retrospective	Examining the equality of resource distribution between Iran provinces in 2014	Proposing suggestions to policymakers to focus more on resource distribution-based health requirements of patients	Not identified
